# Microglia in Neuroinflammation and Neurodegeneration: From Understanding to Therapy

**DOI:** 10.3389/fnins.2021.742065

**Published:** 2021-09-24

**Authors:** Luca Muzio, Alice Viotti, Gianvito Martino

**Affiliations:** Neuroimmunology Unit, Division of Neuroscience, IRCCS San Raffaele Hospital, Vita-Salute San Raffaele University, Milan, Italy

**Keywords:** microglia, brain aging, Alzheimer’ disease, Parkinson’s disease, amyotrophic lateral sclerosis, neuroinflammation, multiple sclerosis, neurodegeneration

## Abstract

Microglia are the resident macrophages of the central nervous system (CNS) acting as the first line of defense in the brain by phagocytosing harmful pathogens and cellular debris. Microglia emerge from early erythromyeloid progenitors of the yolk sac and enter the developing brain before the establishment of a fully mature blood–brain barrier. In physiological conditions, during brain development, microglia contribute to CNS homeostasis by supporting cell proliferation of neural precursors. In post-natal life, such cells contribute to preserving the integrity of neuronal circuits by sculpting synapses. After a CNS injury, microglia change their morphology and down-regulate those genes supporting homeostatic functions. However, it is still unclear whether such changes are accompanied by molecular and functional modifications that might contribute to the pathological process. While comprehensive transcriptome analyses at the single-cell level have identified specific gene perturbations occurring in the “pathological” microglia, still the precise protective/detrimental role of microglia in neurological disorders is far from being fully elucidated. In this review, the results so far obtained regarding the role of microglia in neurodegenerative disorders will be discussed. There is solid and sound evidence suggesting that regulating microglia functions during disease pathology might represent a strategy to develop future therapies aimed at counteracting brain degeneration in multiple sclerosis, Alzheimer’s disease, Parkinson’s disease, and amyotrophic lateral sclerosis.

## Introduction

Microglia are resident immune cells of the central nervous system (CNS) that belong to the population of primary innate immune cells (Davalos et al., 2005; Nimmerjahn et al., 2005). Microglia are long-lived cells that arise from a transient hematopoietic wave of erythromyeloid precursor cells emerging in the yolk sac (Ginhoux et al., 2010; Li and Barres, 2018). The healthy adult CNS does not receive further precursors from post-natal hematopoiesis, so these early progenitors can sustain microglia turnover during their lifetime (Ginhoux et al., 2010). Microglia can also regenerate shortly, as shown in several studies in which these cells were pharmacologically or genetically ablated in the CNS of mice (Waisman et al., 2015). Colony-stimulating factor 1 (CSF-1) is a hematopoietic cytokine that exerts a crucial role in the activity, survival, and maintenance of microglia. Colony-stimulating factor 1 receptor (CSF1R) is the receptor for interleukin (IL) 34 and CSF-1 (Green et al., 2020). The conditional deletion of *Csfr1* in microglia leads to a substantial depletion of these cells (Elmore et al., 2014). The pharmacological inhibition of CSFR1 depletes microglia, although such manipulation has relevant effects on peripheral immune cells (Lei et al., 2020).

The innate immune responses are considered the first line of defense against invading pathogens. Therefore, the activation of microglia is protective for the brain. However, sustained or chronic activation of microglia can lead to irreversible CNS damage. Indeed, persistent inflammation in the brain affects neuronal plasticity, impairs memory, and is generally considered a typical driver of tissue damage in neurodegenerative disorders. Recent observations add further levels of complexity to the comprehension of microglia-mediated mechanisms affecting the brain. Comparing microglia signature in neuroinflammatory vs. neurodegenerative disorders suggests the existence of subsets of activated microglia – that can be defined by common cell surface markers – expressing heterogeneous cytokines that might contribute to the tissue damage vs. repair in different ways (Ajami et al., 2018).

Microglia operate as safeguards of the CNS, scanning the environment for danger cues and/or invading pathogens: being regularly distributed throughout the CNS, like watchmen, they undergo activation by local danger cues. Microglia actively adapt cell morphology in response to these signals, by increasing soma size and retracting their thin cytoplasmic processes (Szalay et al., 2016). In neurodegenerative processes, chronically activated microglia do release inflammatory cytokines, such as tumor necrosis factor α (TNF-α), IL-6, and IL-1β, reactive oxygen species (ROS), and excitotoxins, including glutamate. Via the release of such molecules, microglia might exert both detrimental and protective effects depending on the microenvironment characteristics. Among neuroprotective functions, microglia show the ability to clear apoptotic cells and to release neurotrophic factors and growth hormones in the extracellular space (Hinks and Franklin, 1999).

To promptly respond to local pathogenic cues, microglia are equipped with toll-like receptors (TLRs) that are transmembrane receptors featured by an extracellular leucine-rich repeat domain that detects pathogens-associated molecular patterns (PAMPs) or damage-associated molecular patterns (DAMPs) (Piccinini and Midwood, 2010; Matzinger and Kamala, 2011). Microglia constitutively express a wide array of TLRs (TLR1-9) as shown in rodent and human brains (Bsibsi et al., 2002; Olson and Miller, 2004). The activation of downstream TLRs pathways leads to the production of pro-inflammatory cytokines or to the production of type I interferons (IFN-I), which induces the release of IFN-β and chemokines, such as C-C motif chemokine ligand (CCL) 5 and C-X-C motif chemokine ligand 10 (CXCL10) (Kawai and Akira, 2010).

Microglia increase the rates of proliferation in almost all the neurodegenerative disorders in which these cells have been investigated. This is probably a common feature of microglia that associates with the ability of these cells to secrete a wide array of cytokines and chemokines. However, the exact response and the contribution of these cells to neurodegeneration are probably age- and context-dependent. Genetic studies attempted to relate microglia to the pathobiology of several neurological disorders. Although such results are not exhaustive, they offer intriguing indications that directly or indirectly create functional links between microglia and neurodegenerative disorders. Recent work in multiple sclerosis (MS), analyzing data from 15 genome-wide association studies (GWASs) of MS, identified new gene variants and observed enrichment for MS genes in human microglia (International Multiple Sclerosis Genetics Consortium, 2019). The interpretation of data from GWAS in Alzheimer’s disease (AD) is far from an easy task. However, several studies envisaged that inflammation and microglia activation are part of the pathological mechanisms that lead to dementia. Besides fully penetrant mutations, some risk variants encode for genes that enrichened in microglia [see this recent review for a comprehensive description on genetic risk in AD (Sierksma et al., 2020)]. Amyotrophic lateral sclerosis (ALS) disease is markedly heterogeneous both at genetic and phenotypic levels. GWAS studies identified the chromosome 9p21 locus that accounts for nearly half of familial ALS cases (Laaksovirta et al., 2010). A further investigation allowed the identification of a large hexanucleotide expansion in the first intron of a long transcript of C9ORF72 and this repeat segregates with disease in a large cohort of familial ALS patients (DeJesus-Hernandez et al., 2011; Renton et al., 2011). *C9orf72* is widely expressed by myeloid cells, including microglia (Rizzu et al., 2016). A deficit of C9orf72 altered the homeostatic gene signature of microglia while C9orf72-deficient microglia promote synaptic loss and behavioral defects in mice (Lall et al., 2021). Using the largest set of summary statistics from Parkinson’s disease (PD) GWAS a recent study showed significant enrichment of PD risk heritability in open chromatin regions of microglia and monocytes, supporting the importance of these cells in PD pathogenesis (Andersen et al., 2021).

**FIGURE 1:**
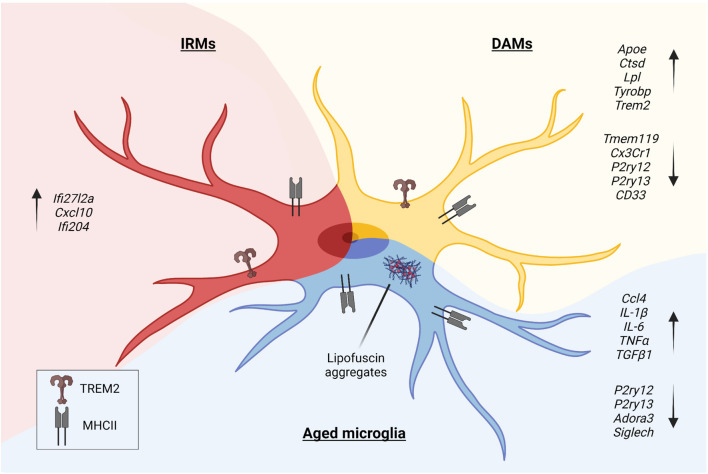
In the pathological context, microglia undergo morphologic and phenotypic changes upon activation. Disease-Associated Microglia (DAMs), Injury-Responsive Microglia (IRMs), and aged microglia represent different activation states, each characterized by a specific transcriptional signature.

Large and massive campaigns of single-cell RNA sequencing of microglia in animal models of CNS diseases allowed the identification and characterization of disease-associated microglia (DAM) (Deczkowska et al., 2018; Figure 1). The induction of the DAM phenotype is far to be completely understood, but it could be the result of recurrent pathways of activation. Indeed, local danger signals are similar in different neurodegenerative disorders and can cause the activation of microglia and the acquisition of the DAM phenotype.

Resting microglia express a specific gene signature that is not shared by other immune cell populations. The list of genes includes some “homeostatic” genes, such as purinergic receptor P2Y12 (*P2ry12*), purinergic receptor P2Y13 (*P2ry13*), transmembrane protein 119 (*Tmem119*), legumain (*Lgmn*), tubulin polymerization promoting protein (*Tppp*), bridging integrator 1 (*Bin1*) and regulator of G protein signaling 10 (*Rgs10*) (Butovsky et al., 2014). However, in the pathological model of MS, namely the experimental autoimmune encephalomyelitis (EAE), microglia downregulate some of these makers, acquiring the expression of a pro-inflammatory signature. On the other hand, some genes belonging to the “microglia signature” are expressed by monocyte-derived macrophages (Grassivaro et al., 2020). Therefore, these results indicate a degree of plasticity of myeloid cells during the pathology, thus blurring the border between microglia and macrophages (Grassivaro et al., 2020). It is important to note that only CNS-infiltrating macrophages acquired the capability to express part of the “microglia signature,” indicating that immune cells adapt and change their phenotype over time in response to local signals that probably arise from neurons or astrocytes (Grassivaro et al., 2020). These local cues can be instructive for microglia as well as for myeloid cells that infiltrate the CNS. The study of this plasticity is fundamental to understand part of the pathobiology of neurodegenerative disorders.

Acute and chronic inflammatory CNS disorders are either characterized by the primary inflammation that leads to secondary neurodegeneration (for example MS, spinal cord injury, brain trauma, and stroke), or by the primary neurodegeneration that is accompanied by secondary reactive inflammation (for example AD, ALS, PD, epilepsy, and Huntington’s disease). In this review, we will focus on the role of microglia in neurodegenerative disorders in which inflammation is a reactive process to neuronal damage.

## Microglia and Aging

Longevity has dramatically increased worldwide over the last decades, and the number of individuals aged ≥65 years is expected to become more than double in the next 30 years, with aged people reaching 16% of the total population in 2050 (United Nations Department of Economic and Social Affairs, Population Division, 2020). Aging is a complex process that involves senescence, a gradual loss of homeostasis in virtually all organs, and inflammation. Accordingly, hallmarks of aging are classified as *primary*, *antagonistic*, and *integrative*, and include nuclear and mitochondrial DNA damage, shortening of telomeres, epigenetic alterations, cellular senescence, stem cell exhaustion, inflammation, and loss of proteostasis (Lopez-Otin et al., 2013). Aging associates with deterioration, leading to age-related pathologies that involve every organ and system. In the brain, this phenomenon is progressive, and it is featured by a functional decline that parallels with cognitive impairments. Brain aging is a multifactorial process that results in irreversible changes in cerebral tissue integrity and affects both structural and functional connectivity in neurons (Damoiseaux, 2017). Besides genomic instability, another aspect of brain aging is oxidative stress, which results in nuclear and mitochondrial damage, and lipid peroxidation (Mattson and Arumugam, 2018).

Neurodegenerative diseases, including AD, ALS, and PD, are leading contributors to worldwide disability. Neurodegeneration in these disorders affects neurites, increases rates of apoptosis, causes loss of proteostasis in neurons, as well as induces immune-related alterations in the CNS (Kostic et al., 1997; Masliah et al., 1998; Matarin et al., 2015). Processes sustaining brain aging and those that perpetuate neurodegeneration have been investigated according to a *neurocentric* view for many years. However, cogent experimental evidence deriving from transcriptomic profiling of the CNS, genome-wide association studies, and the use of animal models of CNS diseases, indicates that non-neuronal cells, such as myeloid cells, might play a more prominent role in aging and neurodegeneration. Myeloid cells, including microglia, namely the tissue-resident macrophages of the brain, perivascular macrophages, and monocyte-derived macrophages, express a pattern of risk-genes that can influence the progression of several CNS disorders (Jones et al., 2010; Lambert et al., 2013).

Like neurons also microglia age, therefore these cells are subjected to selective age-dependent alterations. Microglia are the first line of defense in the CNS, being these cells vigilant to pathological alterations occurring in neurons. During the process of aging several pathways linked with immune-vigilant functions are altered in microglia (Angelova and Brown, 2019). These pathways could also be activated in pathological conditions; therefore, it is hard to discriminate between pathways resulting from the pathological activation of microglia and pathways that are activated by the physiological process of aging.

In the pathological context, microglia undergo specific phenotypic changes, as shown by studies that investigated human and animal models of brain disorders. DAM (Keren-Shaul et al., 2017), injury-responsive microglia (IRM) (Hammond et al., 2019), and aged microglia (Safaiyan et al., 2016) depict different states of activation of microglia (Figure 1). DAM downregulate a subset of genes featuring the homeostatic microglia, such as *P2ry12*, *P2ry13*, C-X3-C motif chemokine receptor 1 (*Cx3cr1*), CD33 molecule (*CD33*), and *Tmem119* (Butovsky et al., 2014), and upregulate genes involved in lysosomal, phagocytic, and lipid metabolism pathways, such as apolipoprotein E (*Apoe*), cathepsin D (*Ctsd*), lipoprotein lipase (*Lpl*), transmembrane immune signaling adaptor TYROBP (*Tyrobp*), and triggering receptor expressed on myeloid cells 2 (*Trem2*) (Lambert et al., 2013; Brioschi et al., 2020). Interestingly, TREM2 signaling in microglia sustains the age-dependent expansion of these cells, their ability to skew the transcriptional signature, and it is probably involved in processes leading to neuronal loss that occurs during physiological aging (Linnartz-Gerlach et al., 2019). Single-cell transcriptomics of microglia obtained from aged and young mice further sustained the concept that specific clusters of microglia emerge during the process of aging. Young and aged microglia differ for at least two distinct clusters that appear in the aged brain. The study that identified IRM showed that these cells belong to a cluster displaying a unique upregulation of interferon responsive genes, such as Interferon alpha-Inducible protein 27-like protein 2A (*Ifi27l2a*), interferon activated gene 204 (*Ifi204*), and *Cxcl10*. It has been shown that, within the IRM cluster, microglia differentially regulate specific genes, like baculoviral IAP repeat containing 5 (*Birc5*), *Ccl4*, *Cxcl10*, and *Apoe*, indicating the existence of further subpopulations with selective transcriptional programs involved in the regulation of the inflammatory state and cytokine signaling. Interestingly, with injury and aging, microglia partially re-express markers belonging to developmental subpopulations (Hammond et al., 2019).

A major contributor to the process of aging is cellular senescence. Senescent cells are mainly characterized by a permanent cell cycle arrest (Krenning et al., 2014) as well as by a senescence-associated secretory phenotype (SASP), which is composed of a complex array of signals that includes inflammatory factors (Acosta et al., 2013; Chen et al., 2015). Under homeostatic conditions, young microglia are composed of self-sustaining and proliferating cells (Askew et al., 2017; Fuger et al., 2017). Adult microglia also self-sustain, combining a slow apoptotic flux with a constant cell division process that occurs stochastically in the CNS (Askew et al., 2017; Tay et al., 2017). In humans, this slow turnover replaces 0.08% cells per day (Reu et al., 2017); in rodents, it maintains the density of this population around 5% of the total cell number (Pelvig et al., 2008). Microglia are considered slow dividing, long-living cells (Askew et al., 2017) and whether or not these cells could undergo a senescence-mediated arrest of cell proliferation is a matter of debate.

Using positron emission tomography (PET) in humans to measure changes in the density of the translocator protein (TSPO) system, a study demonstrated that densities of activated microglia tend to increase in healthy aged brains (Gulyas et al., 2011). However, a subsequent study that adopted a second-generation TSPO radioligand, failed to find a significant relationship between age and increased TSPO density in the human brain (Suridjan et al., 2014). Histopathological studies in human brains reported conflicting results about microglia cell numbers and density during the process of aging. One study reports increasing numbers of activated microglia in several sectors of the hippocampus from non-demented elderly adults (DiPatre and Gelman, 1997); while a subsequent investigation that used stereology methods to quantify microglia in the brain failed to detect a substantial increase of these cells in aged brains (Pelvig et al., 2008). The study of marmoset brains showed that the total number of microglia did not change between young and aged brains. However, a substantial decrease of resting microglia and a concurrent increase of dystrophic microglia featured the old brains (Rodriguez-Callejas et al., 2016). Similarly, the study of microglia in the visual cortex (area 17) of young and aged monkeys did not reveal any difference in terms of microglia numbers and density (Peters et al., 2008). Studies in rodents also reported contrasting results. A study in rats showed more hippocampal microglia in aged rats than in young controls, while no differences in microglia numbers were observed in other brains regions. In this study, the repopulation efficiency upon PLX3397 (i.e., a pharmacological inhibitor of CSF1R) treatment was also estimated. Aged rats did not fully recover microglial cells number during repopulation as well as these cells express more cytokines than young microglia (Yegla et al., 2021). The study of young and aged prefrontal cortices of rats did not reveal any difference in terms of cell number, while it has been observed a shift in microglia volume in aged animals (Chan et al., 2018). The study of the mouse hippocampus of young and aged brains showed a significant increase of microglial cells number in aged females but not in aged males (Mouton et al., 2002). On the other hand, another study showed that microglia remain remarkably stable throughout life in all brain areas, except for the thalamus, where microglia cell number is increased with aging (Askew et al., 2017). Thus, microglia seem to exhibit a regional phenotypic diversity in aged rodents.

A greater induction in the expression of immune-amplifying genes occurs in microglia of the cerebellum, while the appearance of this gene signature is less evident in aged microglia of the cerebral cortex (Grabert et al., 2016).

Therefore, variation of microglia cell density in the aged brain is still an open question although it is possible that some differences observed in aged brains may be regionally confined or could depend on techniques used to visualize and to score microglia or on the sex of animals used in these studies.

Aged microglia can exhibit a dystrophic appearance, which is characterized by an increased volume of the soma, the shortening and the fragmentation of cytosolic protrusions (Streit et al., 2004). Dystrophic microglia diverge from activated microglia, although both cell types might express inflammatory signals. In elderly human brains besides dystrophic microglia, many cells show the classical amoeboid cell morphology that several studies associate with activated microglia. These cells express CD68 molecule and the human leukocyte antigen-DR (HLA-DR), which is a class II major histocompatibility antigen (Mattiace et al., 1990; Sheng et al., 1998). Aged rodents display microglia with an activated morphological phenotype (loss of ramifications and amoeboid cell morphology), although these brains lack the dystrophic microglia observed in aged human and non-human primate brains (Streit and Xue, 2010).

The accumulation of lipofuscin within the cytoplasm is commonly observed in aged neurons (Gray and Woulfe, 2005). However, lipofuscin can also be detected in senile rat brains microglia (Singh Kushwaha et al., 2018). Interestingly, aged microglia featured by lipofuscin display a substantial reduction of process motilities (Damani et al., 2011). Transcriptome profiling of young and aged human microglia showed that aging of microglia is associated with reduced expression of many genes involved in actin dynamics, sensor surface receptors, and cell adhesion molecules (Galatro et al., 2017), further corroborating the idea that the process of aging affects microglia process motility (Damani et al., 2011; Figure 1).

Like other immune cells, microglia can release a broad plethora of signals in response to tissue damage. Some of these signals are pro-inflammatory, such as IL-1β, TNF-α, IL-6, IL-12, IL-15, IL-17, Ccl2, and Ccl4, while others are anti-inflammatory such as IL-4, IL-8, IL-10, transforming growth factor (TGF) α, and TGFβ. Aged microglia isolated from the brain of p7.2fms-EGFP mice – i.e., transgenic mice that express the GFP under the promoter CSF1Ra (Sasmono et al., 2003) – express higher IL-1β, TNF-α, IL-6 levels than young microglia, although aged microglia are also able to increase TGFβ1 levels (Sierra et al., 2007). Whole transcriptome analysis of young and aged microglia revealed that aged microglia downregulate pathways that have been associated with neurotoxicity, while pathways associated with neuroprotection are upregulated (Hickman et al., 2013; Figure 1). However, aged microglia had impaired induction of IL-4 receptor α (IL-4 Rα) in response to worse functional outcomes that occur in the damaged CNS (Fenn et al., 2014). Therefore, aged microglia display a substantial alteration of the cell morphology that probably reflects changes in gene expression; these cells are more prone to release inflammatory cues and thus they might sustain the process of *inflammaging* which is a chronic low-grade inflammation that arises with the aging of the brain.

## Microglia in Progressive Multiple Sclerosis

Multiple sclerosis is a chronic neurodegenerative, inflammatory disease of the CNS characterized by the formation of demyelinating lesions. It predominantly affects young adults, and the prevalence of the disease varies from high incidence in North America and Europe (>100/100,000) to low rates in Eastern Asia and Sub-Saharan Africa (2/100,000) (Leray et al., 2016; Kobelt et al., 2017). The Atlas of MS of the National Multiple Sclerosis Society reports that up to 2.8 million people worldwide have MS, with nearly one million in the United States.

The etiology of MS is still unknown, although it is largely conceived that MS is a complex disease in which many genes can modestly increase the susceptibility, possibly cooperating with environmental factors such as ultraviolet B light (UVB) exposure, Epstein–Barr virus (EBV) infection, obesity during the adolescence, commensal microbiota, smoking, and low levels of vitamin D (Ramagopalan et al., 2010). For a long time, MS has been considered a T-cell-mediated autoimmune disorder, although recent advancement of B-cell therapies demonstrates the key role exerted by these cells in the progression of the disease (Greenfield and Hauser, 2018).

The main clinical presentations of MS predominantly involve optic neuritis and brainstem, or spinal cord. About 85% of patients display the so-called relapsing-remitting form of MS (RRMS), which is featured by relapses related to inflammatory episodes affecting the CNS, followed by periods (varying from months to years) of quietness (remissions). The remaining 10–15% of patients display a neurological disability that starts soon after the appearance of first clinical symptoms and increases progressively over time without relapses or remissions (primary progressive MS, PPMS) (Trapp and Nave, 2008). MS can evolve during its progression. Indeed, in approximately 55–65% of patients, 10–15 years after the onset of the early symptoms, RRMS turns into a secondary progressive form of MS (SPMS) (Lublin et al., 2014).

Neurodegeneration occurs since the beginning of the disease, while the presence of inflammatory infiltrates in peri-venular regions – i.e., the pathological hallmark of MS – dominates the early phase, becoming less frequent in progressive forms of MS. This is the reason why therapies targeting the immune system and inflammation are effective in RRMS but not in SPMS (Wiendl and Hohlfeld, 2009).

Inflammatory infiltrates comprehend major histocompatibility complex (MHC) class I restricted CD8+ lymphocytes, B cells, plasma cells, and monocytes/macrophages (Lassmann, 2013). These inflammatory lesions boost demyelination in the white and the gray matter. Furthermore, infiltration of immune cells triggers the activation of both infiltrating and CNS-resident myeloid cells. At the begging of the pathological process, myelin sheets and oligodendrocytes are damaged, while axons and neurons are partially spared. However, with the chronicity of the disease, we have a substantial axonal loss which leads to irreversible neurological deficits (Bjartmar et al., 2000).

A large body of experimental evidence indicates that microglia are involved in MS, although the exact degree of this involvement and how mechanisms depending on microglia can cause tissue damage are still unknown (Konjevic Sabolek et al., 2019). As stated above, MS is a complex disorder, involving a broad number of cell types that can operate both detrimental and protective functions. For example, some extra-parenchymal CNS-resident myeloid cells that are located within the cerebrospinal fluid (CSF) compartment, in the leptomeninges, and subarachnoid and perivascular spaces are certainly activated during the neuroinflammation. They include some dendritic cells and subpopulations of macrophages that have been named border-associated macrophages (BAMs) (Goldmann et al., 2016). These cells are strategically located at the interface between the peripheral environment and the CNS parenchyma and therefore might exert surveillance roles.

The study of MS brains revealed that microglia activation is a common feature in this disorder. Patients with a progressive course of the disease display chronic active lesions with microglia activation usually observed at the lesions’ rim, while such activation has not been observed in inactive lesions (Frischer et al., 2009). Using PET to measure TSPO in the brain of MS patients, it has been observed that microglia activation correlates with disability and prognosis in progressive patients but not with the disability in patients with RRMS (Giannetti et al., 2014). In principle, this observation can be explained considering the presence of a compartmentalized inflammation in the brain of SPMS patients that can sustain a long-lasting activation of regional clusters of microglia. A recent study compared datasets obtained from single-cell RNA sequencing of murine microglia with the transcriptome of CD45+ cells isolated from MS brains. This study identifies unique transcriptomic profiles of microglia in active lesions’ biopsies from patients that are in the early stages of MS (Masuda et al., 2019). The latter study also highlights the presence of phagocytic microglia in MS tissues. Moreover, phagocytic microglial cells have been identified in post mortem white matter biopsies from SPMS patients by single-cell mass cytometry by time of flight (CyTOF)-mediated analysis (Bottcher et al., 2020).

As stated above, MS is a complex disorder that occurs only in humans, therefore none of the existing animal models can recapitulate the extensive variety of clinical, immunological, and pathological features of the disease (Constantinescu et al., 2011). However, EAE is the most used MS pre-clinical immune-mediated experimental tool that scientists have used to dissect pathological mechanisms of MS and to set up therapeutic strategies. The neuroinflammation and the subsequent demyelination that occur in the CNS of animals arise in response to antigen immunization. With a different degree of efficiency, it is possible to induce the EAE model in several vertebrates, using different experimental approaches, although rodents are commonly used in many pre-clinical studies. For example, EAE in mice is obtained immunizing animals with CNS-related antigens that are administered in a strong adjuvant, usually the complete Freud’s adjuvant. The combination of the antigen used to induce the disease and the mouse strain determines the disease course (Baker and Amor, 2014). The most popular and widely used model involves C57BL/6 mice and a short peptide that contains the sequence (amino acids 35–55) of the myelin oligodendrocytes glycoprotein (MOG). In this EAE model, mice develop a monophasic chronic disease that relies on the generation of encephalitogenic T-helper (Th)-1 and Th-17 types of CD4 cells. They cross the blood–brain barrier (BBB), inducing inflammation and ascending paralysis that associate with axonal degeneration and limited primary demyelination (Amor and Baker, 2012). In this strain, the greater severity of the disease has been observed in females (Papenfuss et al., 2004).

The value offered by this model is notable, therefore there is a long list of studies dealing with microglia/macrophages activation in neuroinflammation, using the EAE experimental paradigm (Muzio et al., 2007).

Pharmacological or genetic inhibition of microglia activation is protective and attenuates the EAE severity (Heppner et al., 2005; Bhasin et al., 2007; Goldmann et al., 2013). Similarly, CSF1R inhibition by PLX5622 ablates microglia and macrophages, inducing a substantial reduction of EAE severity (Nissen et al., 2018). These results suggest that microglia exert a detrimental role in EAE, contributing to demyelination and axonal loss. However, recent findings indicate that microglia can also be protective in neuroinflammation. As shown by Tanabe et al. (2019), the pharmacological depletion of microglia by CSF1R inhibition in a specific EAE model that recapitulates the typical progressive phase of SPMS exacerbates demyelination and axonal damage. These contradictory results could be explained considering the existence of different subpopulations of microglia that exert different functions. For example, an interesting study shows the existence of a population of microglia that is pro-inflammatory and detrimental and promotes demyelination. However, soon after the death of these cells, repopulating microglia have regenerative functions and support remyelination (Lloyd et al., 2019). This heterogeneity is also shown in a study that describes the presence of microglia expressing the anti-inflammatory cytokine IFN-β in EAE mice. These cells orchestrate phagocytosis of myelin debris, facilitating remyelination (Kocur et al., 2015). A recent study identified a population of CD11c+ microglia that transiently expand in the mouse brain soon after birth (Wlodarczyk et al., 2017). IL-34 or CSF-1 can induce the expansion of these cells and the treatment of EAE mice with these CSF1R ligands reduces the severity of the disease, indicating that microglia can exert immunomodulatory functions in MS (Wlodarczyk et al., 2018).

On the other hand, the nicotinamide-adenine dinucleotide phosphate (NADPH) oxidase (Nox2) is highly expressed by professional phagocytes, including microglia, and is essential for the activation of these cells. Upon Nox2 activation, microglia increase the chemotaxis of peripheral pathogenic immune cells into the CNS, causing demyelination and axonal damage (Hu et al., 2021). Although this latter study could benefit from a cell-specific KO strategy to investigate Nox2 in microglia, authors clearly show that Nox2-deficient mice display less inflammatory infiltrates and demyelination, as well as that microglia-mediated expression of the pool of cytokines and chemokines, is substantially decreased (Hu et al., 2021).

Microglia activation can release pro-inflammatory cues that affect tissue integrity, but these cells can also deviate from this phenotype acquiring new features and therefore contributing to tissue repair and regeneration. Because of this dual phenotype, the molecular mechanisms that can skew pro-inflammatory microglia toward an anti-inflammatory phenotype would have implications for therapies in MS.

## Microglia in Alzheimer’s Disease

Alzheimer’s disease is a progressive neurodegenerative disorder affecting memory, thinking behavior, cognition, language, and the quality of daily life. The main risk factor for AD is aging. The official death certificates recorded more than 100,000 deaths from AD in 2018, making AD the sixth leading cause of death in the United States and the fifth leading cause of death among elderly people (65 years or more), as reported in Alzheimer association report (2020).

From a genetic perspective, AD can be divided into a rare familial form of the disease that accounts for less than 1% of all patients, including the ones with early- and late-onset diseases, and a multifactorial sporadic form of AD, which possibly involves combinations of environmental and genetic causes (Brouwers et al., 2008). Highly penetrant mutations, responsible for rare monogenic forms, were identified in patients carrying mutations in amyloid precursor protein (*APP*) gene (Goate et al., 1991) and presenilin 1 and 2 (*PSEN1* and *PSEN2*) genes (Clark et al., 1995; Rogaev et al., 1995; Brouwers et al., 2008). These rare mutations were found exclusively in early-onset AD patients. Human GWAS identified more than 30 risk loci that are predicted to increase susceptibility for AD. Some of these variants are in or near genes that are expressed by microglia. Among them, the *APOE* gene variant *APOE* E4 and a rare variant of the *TREM2* gene (R74H) associate with AD (Guerreiro et al., 2013; Jonsson et al., 2013).

Two main pathogenic hypotheses have been proposed to explain AD pathobiology. The amyloid cascade hypothesis proposes that AD is caused by the accumulation of extracellular Amyloid β (Aβ) deposits that derive from multiple proteolytic cleavages of APP (Hardy and Allsop, 1991). Instead, the tau propagation hypothesis suggests that intracellular fibrillary aggregates originated by hyperphosphorylated tau affect the cytoplasmic functionality and the axonal transport of neurons leading to their death. The investigation of the natural history of the disease indicates that the deposition of Aβ occurs early in the disease and is followed by tau pathology (Selkoe and Hardy, 2016). The tau pathology is observed initially in specific regions of the brain and then spreads to other areas (Frost et al., 2009). However, recent experiments suggest that amyloids deposition could be not necessarily the trigger of neuronal degeneration. This observation comes from the study of centenarians, who display apparent good cognitive functions while their brains contain several amyloids deposits (Ganz et al., 2018).

Additional stress effectors accumulate along the disease progression concurring to harm neurons. Among them there are depletion of neurotransmitters, mitochondrial dysfunction, oxidative damage, a decline of synapse activity, and inflammation (Querfurth and LaFerla, 2010). About the immune activation and the subsequent neuroinflammation, gene variants that associate with microglia exert a considerable influence on disease progression (Sims et al., 2017). The deposition of Aβ plaques triggers microglia activation, potentially leading to Aβ peptides phagocytosis and promoting the clearance of these toxic species (Hansen et al., 2018).

Alzheimer’s disease has been investigated in rodent models using a long list of transgenic mouse lines that overexpress mutant forms of the human *APP* gene (often the Swedish mutation). The most popular strains are Tg2576-APPSWE, APP23, PDAPP, tgCRND8, and APP-KI (Games et al., 1995; Hsiao et al., 1996; Sturchler-Pierrat et al., 1997; Chishti et al., 2001; Saito et al., 2014). Additional AD animal models overexpress mutant forms of both *APP* and *PSEN1* genes. Among these mouse lines, we find APPPS1, 5XFAD, and 3Xtg-AD (Oddo et al., 2003; Oakley et al., 2006; Radde et al., 2006). Although these transgenic mouse lines express mutant proteins at levels much higher than observed in the human brain, they often do not recapitulate the accumulation of neurofibrillary tangles. In addition, only a few of them shows the widespread neuronal loss that is observed in patients. However, the majority of our knowledge about the role of microglia in AD pathobiology comes from the profiling of postmortem brain tissues of AD patients and the investigation of these animal models. For example, recent studies, performed in human and animal models, analyzed the transcriptome of microglia with single-cell resolution. Such studies provided relevant details about the existence of specific microglia subtypes in AD brains that had been missed in studies using bulk tissue-derived cell populations.

**FIGURE 2:**
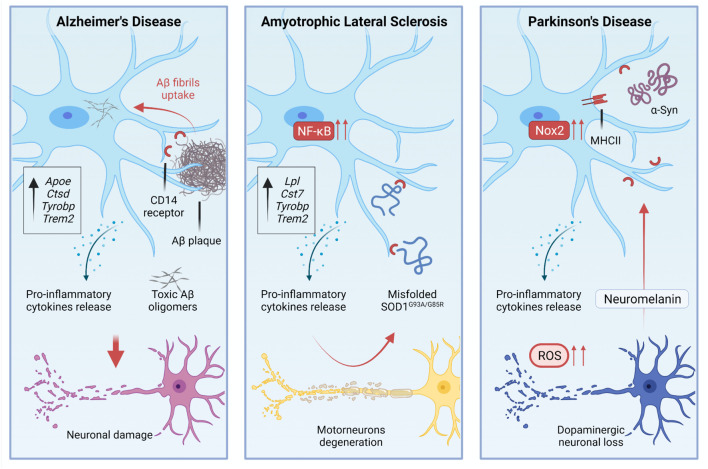
During pathological processes, microglial activation is involved in the disease progression, residing in a chronic state of activation.

The study of microglia in AD models has a long story that started with the observation of a tight association between microglia and Aβ deposits (Frautschy et al., 1998). Such association is quite similar to the association that has been observed in patients (Sheng et al., 1997). The interplay between microglia and Aβ deposits implies the activation of specific gene programs in microglia. For example, CD14 Molecule (CD14) receptors, which are expressed by microglia, interact with fibrils of the amyloid peptide (Fassbender et al., 2004; Figure 2). Single-cell RNA-sequencing of CD45+ cells in 5XFAD mice showed the existence of distinct immune subpopulations in the brain of this popular AD model. Differentially expression gene analysis of these immune cells revealed the existence of a distinctive microglia phenotype that authors indicated as DAM (Keren-Shaul et al., 2017). Interestingly, DAM express some AD risk genes such as *Apoe* (Corder et al., 1993), *Ctsd* (Paz et al., 2015), *Tyrobp* (Pottier et al., 2016), and *Trem2* (Guerreiro et al., 2013; Keren-Shaul et al., 2017). DAM expressing *Trem2* at high levels are in the proximity of Aβ plaques. *Trem2* is relevant for the establishment of DAM. Indeed DAM in 5XFAD mice lacking *Trem2* are almost completely absent (Keren-Shaul et al., 2017; Figure 1). Far to be conclusive, this finding suggests the existence of a subpopulation of microglia in AD mice that activate an intrinsic mechanism to counteract neuronal degeneration and cell death. Of course, understanding the activation and role of these cells could be instrumental to design new therapeutic tools. However, it is also important to note that the predictive validity of 5XFAD mice is still debated. Indeed, AD is a heterogeneous disease in which several players can mediate different detrimental effects that eventually lead to cognitive loss and disease escalation. In the progression of pathological outcomes, 5XFAD mice start to accumulate amyloid deposits very early in their life (Oakley et al., 2006), and this is followed by a selective loss of noradrenergic (Kalinin et al., 2012) and cholinergic neurons (Devi and Ohno, 2010). Thus, to test pathological mechanisms leading to AD, these mice may not be the best choice.

Several studies attempted to modulate microglia in AD models to assess whether this population of cells could change the progression of the disease. Most of the work has been done crossing mice lacking *Trem2* with AD mice. However, some contrasting results emerged from the use of *Trem2* knockout mutants. The inactivation of *Trem2* in APPPS1 mice reduced the number of infiltrating macrophages that associate with Aβ plaque the levels of inflammatory cytokines in the brain and, above all, the accumulation of Aβ deposits. These results suggest that *Trem2* deficiency in AD is protective (Jay et al., 2015). In contrast, experiments based on parabiosis showed that the infiltration of peripheral monocytes in 5XFAD and APPPS1-21 mice is negligible (Wang et al., 2016). In this study, the depletion of TREM2 in AD mice impaired the interactions between microglia and plaques, although it did not alter the total Aβ deposition. Nevertheless, the absence of TREM2 slightly, but significantly increased the number of dystrophic neurites in AD mice (Wang et al., 2016). These results are in line with human genetic studies suggesting that TREM2 loss of function variants promote AD, and with preclinical studies showing that the inactivation of TREM2 in AD mice is detrimental. These results can be explained considering that microglia lacking TREM2 are unable to polarize their cytosolic processes toward the plaque surface and to create a physical barrier that separates Aβ plaques from neurites (Yuan et al., 2016). The overexpression of human *TREM2* in 5XFAD mice can limit the size and the diffusion of amyloid plaques shifting the morphology of plaques from fibrillary – i.e., plaques that induce neurotoxicity – to more compact and inert types as well as the increase of *TREM2* dosage enhanced the phagocytic activity of microglia (Lee et al., 2018; Figure 2). However, what we know about microglia in AD is probably a part of the story as shown by additional studies that investigated microglia in tauopathy. Indeed in a pure animal model of tauopathy, the depletion of microglia arrests the propagation of tau in the brain (Asai et al., 2015).

Recently, single nucleus RNA sequencing of microglia from sporadic AD patients and patients carrying the *TREM2* R47H mutation revealed the existence of a new subset of microglia that authors designed amyloid-responsive microglia (ARM) (Nguyen et al., 2020). ARM are CD163+ and differ from homeostatic and dystrophic microglia. They are conceptually like DAM. Interestingly, ARM responses are reduced in *TREM2* R47H AD brains, suggesting that TREM2 exerts protective functions in AD (Nguyen et al., 2020).

Additional experiments highlight the key role of TREM2 in AD pathobiology. Indeed, a study reported that Galectin-3 (Gal-3), a member of a family of glycan-binding proteins, is a TREM2 endogenous ligand (Boza-Serrano et al., 2019). Gal-3 is involved in several relevant biological processes exerting pleiotropic functions that include: cell adhesion, proliferation, migration, apoptosis, and inflammation (Dong et al., 2018; Tan et al., 2021). Gal-3 is increased in the serum of AD patients (Yazar et al., 2020), as well as it is ten folds increased in microglia of AD brains and 5XFAD mice (Boza-Serrano et al., 2019). Plaque-associated microglia are the predominant cell type expressing Gal-3 in humans and mice. Importantly, Gal-3 inactivation in 5XFAD mice attenuates the whole immune response, including the expression of some genes that belong to the DAM signature (Boza-Serrano et al., 2019). Moreover, 5XFAD/Gal-3KO mice exhibited a substantial reduction of Aβ deposits, indicating that Gal-3 can serve as a regulator of AD-associated pathology (Boza-Serrano et al., 2019). Thus it is not surprising that polymorphisms of the *LGALS3* gene that encodes for Gal-3, associate with a decline in cognitive performance in a large cohort of aged adults (Trompet et al., 2012). The use of innovative techniques for microglia profiling significantly increases our knowledge about the phenotypes that microglia can acquire in pathological conditions, posing the conceptual basis for additional investigations in humans. Single nucleus transcriptome profiling of a relatively large cohort of human AD brains indicates the existence of a specific gene signature for microglia that associates with AD. Genes featuring the DAM signature in AD animal models (Keren-Shaul et al., 2017) are also represented in human AD microglia nuclei, although the human microglia also express a set of exclusive genes that are not seen in the mouse microglia (Mathys et al., 2019).

In a parallel study, single nucleus transcriptome analysis was used to characterize cells captured in the entorhinal cortex of six AD patients. This analysis uncovers five microglia clusters. In some of them, microglia downregulated homeostatic genes, such as *CX3CR1*, *P2RY12*, and genes involved in cell-cell adhesion, lipid response, and G protein-coupled receptor (GPCRs) pathways (Grubman et al., 2019). The single-cell analysis provided in this study captured divergent gene expression variations among different cell types that populate the AD brain. This is the case of the risk gene *APOE*, which is downregulated in AD oligodendrocytes progenitors and astrocytes while upregulated in specific clusters of AD microglia (Grubman et al., 2019).

A further study based on single cells sequencing of microglia obtained from two cortical regions of human AD brains confirmed the existence of several microglia clusters. As expected, some of these clusters express genes that commonly associate with homeostatic microglia, while others are enriched in genes involved in interferon response and antigen presentation. However, in this study, genes featuring the mouse DAM microglia (Keren-Shaul et al., 2017) are shared by multiple microglia clusters, suggesting that the human microglia diverge from the mouse microglia (Olah et al., 2020).

Recently, microglia gene expression has been profiled in AD brain regions displaying Aβ pathology and in regions in which there is a combination of Aβ and tau pathology. The technology used in this work is the nucleus RNA sequencing but the population of cells assayed in this study was depleted of neuronal nuclear antigen (NEUN)+ and oligodendrocyte transcription factor 2 (OLIG2)+ nuclei; thus the goal of this study is to capture small groups of microglia subtypes (Gerrits et al., 2021). The comparison of gene expression between these two regions revealed the existence of two distinct AD-associated populations of microglia. One subset of microglia associates with Aβ deposition (AD1) while another subset of cells associates with regions displaying hyperphosphorylated tau (AD2). While AD1 microglia share characteristics of activated/phagocytic microglia that are also observed in Aβ-plaques of animal models of AD, AD2 cells express homeostatic genes and some neuron-related genes such as glutamate ionotropic receptor delta type subunit 2 (*GRID2*). These cells might exert tissue-supportive and neurotrophic functions in response to neuronal stress (Gerrits et al., 2021). Decrypting the role of these cells can be instrumental in the design of new therapeutic strategies to counteract neuronal degeneration.

The central role of microglia in AD provided by the recent literature expanded our knowledge about mechanisms leading to AD pathobiology and allowed the development of new therapeutic tools. A recent study tested the effects of a human TREM2 agonistic antibody (AL002c) in AD models that were generated crossing mice lacking the mouse *Trem2* gene and expressing either the common human *TREM2* variant or the R47H variant with 5XFAD mice. The TREM2 antibody can cross the BBB and *in vitro* can activate the human TREM2. The administration of AL002c to AD models slightly, but significantly, reduced filamentous Aβ plaques and neurites dystrophy, promoting phagocytic activity of microglia (Wang et al., 2020). Interestingly, the authors of this study also demonstrated the safety of AL002 treatment in a phase 1 clinical trial performed in healthy adult subjects (Wang et al., 2020).

Another manipulation of microglia involving the injection of IL-33 in APP/PS1 mice increased the mobility of microglia and induced the expression of genes associated with antigen presentation, actin filament organization. However, IL-33 did not affect the proportion of DAM observed in the brain of AD mice. IL-33 treatment induced a sub-population of microglia to acquire a specific phenotype (IL-33RM) with higher Aβ phagocytic capacity and therefore this manipulation increased the Aβ clearance in APP/PS1 mice (Lau et al., 2020).

As stated at the begging of this section, aging is the main risk factor for AD, largely affecting the functionality of the brain. There is a loss of general trophic support from non-neuronal cells. However, aging affects the immune system as well. The senesce of the immune system leads to chronic inflammation and in AD the immunosenescence is accompanied by alterations of the balance between adaptive and innate immune functions with a clear shift toward the innate response (van der Willik et al., 2019). Many pieces of evidence based on AD animal models strongly suggest that neuroinflammation has a predominant role in the disease’s onset and progression. We highlighted the key role of TREM2 in AD and the existence of diverse microglial subpopulations that differentially respond to pathological stimulation. However, contrasting results emerged while evaluating the role of TREM2 and microglial involvement in AD mouse models. Limitations of current AD murine models are the absence of clear neurodegeneration and human-like tau pathology, thus being able to only partially recapitulate AD pathophysiology. From a therapeutic perspective, it would be interesting to deepen the role of disease-specific microglial phenotypes during the pre-disease period and AD progression to design immunotherapy that modulates inflammation.

## Microglia in Amyotrophic Lateral Sclerosis

Amyotrophic lateral sclerosis (also known in the United States as Lou Gehrig’s disease) is a degenerative disorder affecting motor neurons (MNs) of the cerebral cortex, brainstem, and spinal cord. ALS is a fatal disease, leading to the death of patients with a median survival from diagnosis of 30 months (Rowland and Shneider, 2001). ALS occurs as sporadic in about 90% of patients, while the remaining 10% display a positive familial ALS (Sabatelli et al., 2013). A list of 25 genes have been associated with the disease, such as the Cu/Zn superoxide dismutase (*SOD1*), TAR DNA binding protein 43 (*TARDPB*, encoding TDP-43), fused in sarcoma (*FUS*), and chromosome 9 open reading frame 72 (*C9ORF72*) (Kim et al., 2020).

About ALS pathogenic mechanisms, there is a consensus about the existence of either cell-autonomous (mitochondrial dysfunctions, RNA metabolisms, protein aggregation, axonal transports), or extrinsic factors that depend on non-neuronal cells like microglia and astrocytes (Nagai et al., 2007).

Mechanistically, this concept was first demonstrated on chimeric mice carrying a mixture of cells expressing the mutant form of *SOD1* gene (SOD1^G93A^) (Rosen et al., 1993; Gurney et al., 1994) and non-neuronal cells expressing the wild-type form of the *SOD1* gene. The presence of wild-type non-neuronal cells significantly extended the survival of mice reducing axonal degeneration and MNs loss (Clement et al., 2003). Similarly, lowering the expression levels of the mutant form of the *SOD1* gene (SOD1^G37R^) within microglia extended the survival of mice (Boillee et al., 2006). Although these mice lived longer, they eventually developed the disease, suggesting that pathological processes leading to cell death are still embodied in MNs.

Motor neurons death and the pathological cascades associated with their degeneration engage inflammation as shown in blood and CSF samples of ALS patients (Tateishi et al., 2010). Microglia activation has been observed in humans, as demonstrated by PET analysis of a small cohort of patients. This study revealed the existence of a diffuse microglial activation in both motor and extra-motor cerebral regions (Turner et al., 2004). However, there are also studies reporting reactive microglia only in subsets of patients (Spiller et al., 2018; Tam et al., 2019), while other works showed that microglia activation is a common trait in ALS (D’Erchia et al., 2017; Dols-Icardo et al., 2020). A general limitation of these studies is the small number of cases that have been investigated. Gene expression in glial cells exhibits a shift during the physiological process of aging (Soreq et al., 2017) and therefore different ages of patients enrolled in these studies could hamper the interpretation of these results. Furthermore, several findings derive from the examination of post-mortem tissues and therefore they can only provide indications about what occurs in the brain at the final stage of the disease. However, studies involving animal models of ALS further delved deeper into microglia activation in ALS, providing the ground for the manipulation of these cells in animal models of the disease.

Early in the disease process, microglia from SOD1^G93A^ mice express mRNAs that are related to the M2 activation profile. This phenotype is due to the activation of myeloid cells by Th2 cytokines (IL-4, IL-10) and differs from the classic activation profile (M1) because M2 cells release cytokines with anti-inflammatory activity (IL-4, IL-10, and IL-13) and neurotrophic factors such as brain-derived neurotrophic factor (BDNF), instead of pro-inflammatory signals (Gordon, 2003; Tang and Le, 2016). Near the final stage of the disease, microglia from SOD1^G93A^ mice switch from the M2 phenotype to the M1 phenotype, starting to exhibit phagocytic activity and to produce pro-inflammatory mediators, including ROS and nitric oxide (NO), IL-1β, IL-6, TNF-α (Liao et al., 2012).

However, the M1/2 paradigm is likely to be an oversimplification of the true microglia phenotype (Ransohoff, 2016). Indeed, the analysis of RNA sequencing data from microglia sampled from pre-symptomatic, symptomatic, and end-stage SOD1^G93A^ mice showed that starting from the onset of the disease, ALS microglia differ from control microglia, but also that SOD1^G93A^ microglia display a significant induction of potentially neuroprotective and neurotoxic factors concurrently during disease progression (Chiu et al., 2013). Additional efforts to study spatiotemporal dynamics of microglia activation in SOD1^G93A^ mice revealed that microglia dysfunctions may occur before the onset of the disease (Maniatis et al., 2019).

Single-cell RNA sequencing of CD45+ cells sorted from SOD1^G93A^ mice showed that a subpopulation of microglia expresses an mRNA signature like what has been described in AD-related DAMs. These cells downregulate homeostatic microglia genes, such as *Cx3Cr1* and *P2ry12* while upregulating *Trem2*, *Tyrobp*, *Lpl*, and Cystatin F (*Cst7*) genes, which are linked to phagocytic and lipid metabolism pathways (Keren-Shaul et al., 2017; Figures 1, 2). The loss of *TREM2* in humans increases the susceptibility to develop early-onset dementia (Hickman and El Khoury, 2014), and a rare variant of *TREM2* increases the risk for late-onset AD (Guerreiro et al., 2013). Since the acquisition of the DAM phenotype relies on *TREM2* expression, we could envisage a protective role of this subset of microglia. However, scoring the spatiotemporal expression of *Trem2* in SOD1^G93A^ mice it has been shown that TREM2-mediated signaling in microglia is an early event that anticipates changes in MNs (Maniatis et al., 2019).

Amyotrophic lateral sclerosis is featured by protein aggregation and misfolding (Rubinsztein, 2006). Misfolded SOD1 mutants released in the extracellular space can induce microgliosis (Urushitani et al., 2006). Indeed, extracellular SOD1^G93A^ and SOD1^G85R^ interact with CD14 on microglia and induce the production of pro-inflammatory mediators. Such activation can be attenuated *in vitro* using TLR2, TLR4, and CD14 blocking antibodies, and it is also attenuated in microglia that lack CD14 expression (Zhao et al., 2010). *TLR4* knockout in the SOD1^G93A^ genetic background extended survival of mice by 2 weeks (Lee et al., 2015), while a prolonged pharmacological inhibition of TLR4 in SOD1^G93A^ mice results in a mild attenuation of MNs degeneration but not in prolongation of survival (Fellner et al., 2017; Figure 2).

These landmark studies indicate that microglia activation in animal models is a hallmark of ALS pathological cascades, and potentially a target for the development of new therapeutic interventions.

Nuclear factor-kappa B (NF-kB) is a master regulator of inflammation driving the expression of pro-inflammatory cytokines and chemokines (Ghosh and Karin, 2002). NF-kB activation occurs in SOD1^G93A^ microglia at the late stages of the disease. The reduction of Inhibitor of nuclear factor-kappa B Kinase subunit beta (IKKβ) levels and thus of NF-kB activity in the SOD1^G93A^ genetic background, extended by 20 days the median survival of mice (Frakes et al., 2014).

The inhibition of CSF1R in SOD1^G93A^ mice preserved locomotion performances and extended the survival of mice by 12%. CSF1R inhibition slightly, but significantly, increased the number of surviving MNs (Martinez-Muriana et al., 2016). However, the elimination of 50% of proliferating microglia in SOD1^G93A^ mice using a transgene that expresses the thymidine kinase (*TK*) gene under the control of CD11b did not extend survival of mice (Gowing et al., 2008).

Besides strategies aiming to kill microglia, the manipulations of these cells with anti-inflammatory cytokines gave interesting results.

IL-4 gene therapy in WT mice skews microglia to express genes associated with yolk sac/embryonic microglia (Matcovitch-Natan et al., 2016; Rossi et al., 2018). IL-4 gene therapy in SOD1^G93A^ mice induces microglia to express M2-associated genes, like arginase 1 (*Arg1*), resistin-like alpha (*Retnla*, *Fizz1*), and chitinase-like protein 3 (*Chil3, Ym1*), and concurs to decrease the expression of pro-inflammatory cytokines. However, the shift of microglial activation observed in SOD1^G93A^ mice treated with IL-4 gene therapy induced a slight, but significant, delay of the onset of the disease, a general amelioration of locomotion performances but neither extends survival in mice nor prevents MNs degeneration (Rossi et al., 2018).

The delivery of neutralizing antibodies raised against IL-10 receptor subunit 1 (IL-10R1) in SOD1^G93A^ mice accelerates disease onset, increases bodyweight loss but does not affect survival. On the other hand, adeno-associated viruses (AAV)-mediated delivery in mice of IL-10 significantly delays the clinical onset of the disease, prevents weight loss, and extends survival, although the effects of IL-10 on MNs survival were not taken into account in this work (Gravel et al., 2016).

Granulocyte-colony stimulating factor (GCSF) is a hematopoietic growth factor that is protective in animal models of acute and chronic neurodegenerative diseases (Diederich et al., 2012). Long-term treatment with GCSF increases the median survival in SOD1^G93A^ mice of 12 days, attenuates microgliosis, and the release of TNF-α, although it does not protect MNs from degeneration (Pollari et al., 2011).

Microglia from SOD1^G93A^ mice exhibit potentiation of the purinergic machinery. Indeed, purinergic receptors P2X_4_ and P2X_7_ (P2RX4 and P2RX7) are substantially up-regulated in this animal model (D’Ambrosi et al., 2009). The constitutive deletion of the P2RX7 in SOD1^G93A^ female mice accelerates the onset, worsens the disease progression but extends survival by 9 days (Apolloni et al., 2013). However, the pharmacological inhibition of P2RX7 at the onset of the disease decreases microgliosis, inhibits the expression of NF-kB, significantly attenuates MNs death but does not have any impact on mice survival (Apolloni et al., 2014).

In conclusion, in the context of ALS, targeting microglia has been the aim of various pharmacological and genetic approaches. Many studies attempted to ameliorate the clinical outcome of SOD1^G93A^ mice, with different results in survival prolongation and MNs protection. Microglia modulation through an anti-inflammatory approach succeeded in delaying the onset of the disease but generally failed to prevent MNs degeneration. Although the interpretation of these data is far from a conclusion, it is reasonable to think that the microglia activation occurring in ALS models seems to be a secondary mechanism that starts in response to neuronal damage. Microglia is unlikely the principal driver of MNs death, although these cells can be manipulated to obtain some beneficial effects in the view of a clinical approach.

## Microglia in Parkinson’s Disease

Described for the first time in 1817 as *shaking palsy*, Parkinson’s disease is the second most common neurodegenerative pathology, affecting around 1% of people aged ≥65 years, with more than six million individuals suffering from PD in 2016 (Dorsey et al., 2018). PD is caused by a chronic and progressive loss of dopaminergic (DA) neurons in the substantia nigra pars compacta (SNpc). From a clinical point of view, PD symptoms involve both the central and the peripheral nervous systems and manifest when the pathological stage is already advanced. The clinical phenotype includes rigidity, resting tremor, bradykinesia, postural instability, and a set of non-motor features, like cognitive impairment, autonomic dysfunction, sleep disorder, and depression (Mastrangelo, 2017).

The exact cause of PD is still unknown, but the risk of developing the pathology seems to result from a complex interplay of genetic and environmental factors, both affecting fundamental cellular processes. Indeed, the etiology of the disease is defined as multifactorial and takes into account genetic variants, environmental exposures, and their impact on brain aging (Kalia and Lang, 2015; Ascherio and Schwarzschild, 2016). Although the prevalence of PD cases is defined as sporadic/late-onset, many studies demonstrated the pathology could be genetically driven and heterogeneous (Polymeropoulos et al., 1996, 1997; Farrer et al., 1998). Indeed, familial Parkinsonism refers to disease forms with either an autosomal dominant or recessive pattern. These forms of the disease account for a small fraction of all PD cases (<5%) and commonly result in early-onset PD.

Genetic forms of PD include gene variants in 23 loci (named with progressive *PARK* acronyms) that have been identified by both Mendelian inheritance patterns and GWAS studies. Mutations in PD-associated genes encoding for the PTEN-induced kinase 1 (*PINK1, PARK6*), Parkin (*PRKN, PARK2*), and protein deglycase (*DJ-1*, *PARK7*) proteins cause autosomal recessive PD and are involved in mitochondrial homeostasis and mitophagy (Abou-Sleiman et al., 2004; Trempe et al., 2013). Additionally, mutant forms of other PD-associated genes can cause an autosomal dominant form of the disease. These genes encode for cytosolic proteins and include leucine-rich repeat kinase 2 (*LRRK2*, *PARK8*) and α-synuclein (*SNCA*, *PARK1*), which are interestingly linked to mitochondrial function modulation as well (Lunati et al., 2018).

Hallmarks of the disease are the aggregation of intracellular inclusions known as Lewy bodies (LBs) and Lewy neurites (LNs), mainly composed of aggregated forms of the α-synuclein protein, and the progressive loss of DA neurons in the SNpc (Spillantini et al., 1997). As the pathology evolves, the nigral damage is followed by extensive extra-nigral alterations, involving the dorsal motor nucleus of the glossopharyngeal and vagal nerves, many sub-nuclei of the thalamus and amygdala, up to the neocortex in case of severe damage (Braak and Braak, 2000).

To date, there is no cure for PD, although some available symptomatic treatments can mitigate disease symptoms in patients, increasing their quality of life. Classical parkinsonism is typically characterized by an excellent and sustained therapeutic response to levodopa (L-dopa) (Carlsson et al., 1957), while other medications, such as dopamine agonists, monoamine oxidase-B (MAO-B) inhibitors, and catechol-*O*-methyltransferase (COMT) inhibitors are also commonly used in the clinical practice (Armstrong and Okun, 2020). Unfortunately, these treatments have some limitations, such as the capability to relieve only some of the symptoms that patients experience in daily life. Moreover, they do not halt neuronal degeneration and usually, they have limited long-term efficacy.

As the clinical phenotype of PD is highly heterogeneous, a variety of animal models is available to investigate different aspects of the disease. The 1-methyl-4-phenyl-1,2,3,6-tetrahydropyridine (MPTP) model is one of the most widely used to recapitulate many hallmarks of PD, both in rodents and in non-human primates. MPTP is a highly lipophilic toxin that can cross the BBB, inducing the degeneration of DA neurons that correlates with motor deficits. To exert its effects, MPTP needs to be converted into its toxic metabolite 1-methyl-4-phenylpyridinium (MPP+) ion. MPP+ is released and then transported into DA cells, where it accumulates into mitochondria, and inhibits the complex-I, leading to ATP depletion and increased ROS production (Dauer and Przedborski, 2003; Jackson-Lewis and Przedborski, 2007). The use of this experimental model largely contributed to investigate mechanisms leading to the degeneration of DA neurons, and highlighted the role of mitochondrial dysfunction and neuroinflammation in PD. Similarly, 6-hydroxydopamine (6-OHDA), an analog of dopamine and norepinephrine, induces cell death through the inhibition of the mitochondrial complex-I, promoting oxidative stress (Schober, 2004). Of note, neurotoxic PD models also include pesticides and herbicides, with a particular emphasis on rotenone and paraquat, as environmental factors able to affect PD risk (Vaccari et al., 2019). In addition to these neurotoxins, the administration of high-dose lipopolysaccharide (LPS) has been used to generate animal models of PD (Deng et al., 2021). An alternative to the use of neurotoxins is the generation of genetic models. Among the different and numerous models used to study PD, that include mutated α-*synuclein*, *Lrrk2*, and *DJ-1*, *PINK1*, and *Parkin* knockout mice, none of them can recapitulate both DA neurons degeneration and α-synuclein aggregation, highlighting the actual complexity of replicating PD pathobiology in animal models (Konnova and Swanberg, 2018).

Microglia activation and inflammation are certainly implicated in PD, as shown by several studies. Although we still do not know the relevance of both processes for the establishment of the disease or its progression (Block et al., 2007; Kannarkat et al., 2013). Several studies showed the presence of reactive microglia within the SNpc, the putamen, hippocampus, trans-entorhinal, temporal, and cingulate cortices of PD patients (McGeer et al., 1988; Imamura et al., 2003), as well as the presence of gliosis in subjects that self-administered MPTP (Langston et al., 1999). These cells express the MHC II proteins and probably acquire the status of pro-inflammatory cells (Figure 2). One of the first experimental studies with the MPTP showed that microglia cell number increased in mice injected with this drug. Moreover, the authors reported a change of microglia cell morphology and concurrently a significant loss of DA neurons (Czlonkowska et al., 1996). Using single-cell laser capture to purify microglia from the SN and the hippocampus (CA1, a region relatively spared in PD) of PD and control brains, a study reported a substantial heterogeneity of SN microglia in comparison with CA1 microglia. In addition, the authors defined pathways that are altered in PD microglia. Among them, there are the inflammation-related aldosterone pathway and ROS metabolisms, although the most interesting pathways observed in PD microglia relate to synaptic transmission and neuronal repair (Mastroeni et al., 2018).

A further step toward a better comprehension of microglia in PD comes from a study that used single nucleus transcriptome analysis to explore cells collected from the SN of PD brains. The authors failed to find an association between PD genetic risk and microglia and concluded that the inflammation might exert a lesser role in PD risk than in AD risk (Agarwal et al., 2020). However, a recent study explored the transcriptome of idiopathic PD brains using single nuclei from frozen ventral sections of human postmortem midbrains. The authors of this study found that PD risk variants are associated with microglia and neurons. They also described a change in cell morphology in midbrain microglia obtained from PD specimens: these cells shortened their branching, acquiring the classical amoeboid cell morphology of activated microglia (Smajic et al., medRxiv^1^).

Numerous studies reported the detection of elevated amounts of pro-inflammatory molecules in SN, striatum, and CSF of PD patients (Blum-Degen et al., 1995; Mogi et al., 1996; Hunot et al., 1999), as well as increased levels of IFN-γ in their plasma (Mount et al., 2007). Along the same lines, high levels of both soluble IL-2 receptor (IL-2R) and TNF-α in blood samples of PD patients were significantly associated with more severe symptoms of the disease (Lindqvist et al., 2012). High levels of IFN-γ and TNF-α persisted in Parkinsonian macaque years after MPTP exposure, suggesting long-lasting activation of microglia in PD (Barcia et al., 2011).

The adaptive immune response has been associated with PD, as a trigger of microglia activation. Indeed, the contribution of fragment crystallizable γ (Fcγ) has been investigated in animal models, showing that immunoglobulin can activate Fcγ receptor (FcγR)+ microglia that affect DA cell survival (He et al., 2002). Neuromelanin is an insoluble complex of melanin, composed of peptides and lipids that is released by damaged DA neurons. PD patients displayed high levels of autoantibodies against melanin. Therefore such autoantibodies can serve as a stimulus for microglia activation, contributing to the self-perpetuating nature of DA neurons loss (Double et al., 2009). Intranigral neuromelanin injections strongly activated microglia, inducing these cells to increase ionized calcium-binding adapter molecule 1 (IBA1), CD16/32 levels, while *in vitro* neuromelanin stimulates chemotaxis and the release of pro-inflammatory signals in the BV2 microglial cell line (Viceconte et al., 2015).

Peripheral T cells can infiltrate the CNS of PD patients (Brochard et al., 2009), and interact with the α-synuclein protein, stimulating microglial activation – i.e., increase of MHC II levels – and eventually concurring to the demise of DA neurons as shown in a rat model (Subbarayan et al., 2020). In addition, a study showed that extracellular aggregated human α-synuclein enhanced the activation of Nox2 in microglia, increasing the release of ROS that eventually affect DA neurons (Zhang et al., 2005; Figure 2). The critical role of Nox2 in the pathogenesis of PD is further confirmed by experiments performed in mice lacking the catalytic gp91^phox^ subunit of this enzyme and subjected to the MPTP treatment. These mice displayed increased DA neurons cell survival and less oxidative damage when compared with their WT littermates (Wu et al., 2003). The pharmacological inhibition of microglial activation by minocycline, a tetracycline derivative, protects the nigrostriatal dopaminergic pathway and mitigates the induction of Nox2 and inducible nitric oxide synthase (iNOS) (Wu et al., 2002). Following the same approach, the administration of MPTP to transgenic mice lacking TNF-α receptors resulted in a substantial reduction of dopaminergic toxicity, indicating that pro-inflammatory cytokines are a component of the pathogenic scenario that leads to neurodegeneration in PD (Sriram et al., 2002).

Several studies suggest the existence of a functional link between α-synuclein and microglia. Indeed, microglia responded to α-synuclein *in vitro* changing the cell size and the morphology and releasing pro-inflammatory signals, including TNF-α (Su et al., 2008). Oligomeric α-synuclein fibrils engage TLR1/2 on microglia leading to the nuclear translocation of NF-kB and the production of pro-inflammatory cytokines (Daniele et al., 2015). In addition, α-synuclein fibrils can activate the NACHT, LRR, and PYD domains-containing protein 3 (NLRP3) in microglia that in turn release the pro-inflammatory cytokine IL-1β (Codolo et al., 2013). The inhibition of NLRP3 with a BBB permeant small molecule in PD models mitigates nigrostriatal dopaminergic degeneration (Gordon et al., 2018). Microglia contribute to the demise of DA neurons in response to rotenone treatment that synergizing with LPS and induces these cells to release toxic ROS (Gao et al., 2003). Therefore, it is tempting to speculate that higher-ordered α-synuclein, spreading throughout the nervous system, can act as DAMP promoting neuroinflammation.

Missense mutations in the *Lrrk2* gene cause a late-onset form of PD that is almost indistinguishable from the idiopathic disease (Paisan-Ruiz et al., 2004). *Lrrk2* is highly expressed by monocytes and microglia. LPS induces *Lrrk2* expression in microglia contributing to the activation of pro-inflammatory pathways. On the other hand, the pharmacological inhibition of LRRK2 attenuates pro-inflammatory activation of microglia in response to TLR4 cascade (Moehle et al., 2012). However, the inactivation of *Lrrk2* in transgenic mice did not affect DA neuron survival in SNpc (Tong et al., 2010). Mice expressing the *Lrrk2* mutation G2019S – i.e., a mutation that has been associated with neurotoxicity – decreased the DA content in the striatum, although did not induce neuronal cell death, suggesting that dysfunctions of dopaminergic signaling occur without degeneration of the nigrostriatal pathway (Li et al., 2010). Therefore, it is likely that the mutation of *Lrrk2* could be an initial hit, which is not sufficient to induce PD. Additional hits, possibly involving microglia or simple physiological aging, can act as triggers to activate pathological cascades leading to the disease.

Microglia lacking Parkin and receiving a challenge with LPS exacerbated the NLRP3 signaling and significantly increased the production of IL-1β and IL-18 (Mouton-Liger et al., 2018). Along the same lines, a single intranigral injection of LPS affects tyrosine hydroxylase (TH)+ neurons, decreasing DA and its metabolites in the nigrostriatal system (Castano et al., 1998). However, the delivery of minocycline in rats also treated with LPS injections reduced pro-inflammatory genes expression and prevented the loss of TH+ neurons (Tomas-Camardiel et al., 2004).

Overall, various studies have improved our knowledge of microglial involvement in PD. Different approaches demonstrated the presence of reactive microglia in PD brains, as well as exacerbated inflammation. These changes might be triggered by the adaptive immune response, as proposed by many *in vitro* and *in vivo* studies, perpetuating DA toxicity. Considering the inflammatory component of PD, it is essential to deepen microglial implications in the disease onset and progression. A better comprehension of the roles of different microglial phenotypes in PD might help in finding drugs targeting neuroinflammation as a future strategy for limiting the spread of PD neuropathology.

## Clinical Applications

At the heart of the unmet needs in the treatment of neurodegenerative disorders is the development of a cure and/or treatment options that can prevent the deterioration of neurons. The study of animal models suggests that microglia can be involved in neurodegeneration and therefore several trials attempt to study/modulate these cells in patients. We are far from soundly elucidating the role of microglia in these disorders, but the abovementioned investigations certainly pave the way for the development of new therapeutic tools having microglia as a primary target. To offer a comprehensive overview of where we are, we analyzed current clinical trials based on treatments involving microglia in neurodegenerative disorders.

We searched for clinical trials using the database *ClinicalTrials.gov*^2^, which is the largest database for clinical trials and includes research studies from over 200 countries worldwide. This systematic analysis is focused to put on the global distribution of these trials as well as assessing whether they are observational or interventional trials. This search was performed in August 2021, using the search term “microglia.” We considered clinical trials involving adults (18–64 years) and older adults (>65 years) and we do not apply any gender bias to the search. We identified 111 trials using these simple search criteria. Among them, 16 trials are dedicated to patients with MS (Table 1). Six trials preferentially enrolled patients with primary and secondary progressive forms of the disease), while three trials are designed to manage both RRMS and progressive MS patients (Table 2). Among these 16 trials, 9 are interventional and 2 of them reported results. One trial reported a dose-escalation phase 1b trial of MOR103 (a neutralizing antibody against granulocyte-macrophage colony-stimulating factor, GM-CSF), in patients with RRMS and SPMS. Although this trial has the limitations featuring phase 1/2 clinical trials, including small samples and limited duration, it reports well tolerability of the treatment with no safety concerns (Constantinescu et al., 2015). One trial investigated the effects of adrenocorticotropic hormone (ACTH) on myelination and microglia/macrophages activation in 15 patients. The authors compared two regimens (one-time treatment versus a monthly treatment) reporting no adverse events in both treatments. However, the relatively small number of participants does not allow to draw definitive conclusions from such a study.

**TABLE 1:** Registered clinical trials on https://clinicaltrials.gov focused on *microglia* and *multiple sclerosis.*

NCT number	Title	Status	Conditions	Study type	Interventions	Phase	Study completion date
NCT04925557	Study to Assess the Efficacy of Mayzent on Microglia in Secondary Progressive Multiple Sclerosis	Not yet recruiting	Secondary progressive multiple sclerosis	Interventional	∙ Drug: Mayzent ∙ Drug: Ocrevus	4	April 30, 2025 (estimated)
NCT03368677	Effect of Teriflunomide Treatment on Microglial Activation in an MS Patient Cohort at Risk of Progression (TERIPET)	Recruiting	Multiple sclerosis	Observational	–	Not applicable	November 2024 (estimated)
NCT04239820	Effect of Cladribine Treatment on Microglial Activation in the CNS (CLADPET)	Recruiting	Multiple sclerosis	Observational	∙ Radiation: Imaging	Not applicable	June 2024 (estimated)
NCT04126772	Multimodal Imaging of MS Reveals the Smoldering Inflammation (PLAQ-MS)	Recruiting	Multiple sclerosis	Observational	–	Not applicable	September 2023 (estimated)
NCT03134716	Role of Microglia in the Pathogenesis of Progressive Multiple Sclerosis (PROMS)	Active, not recruiting	Multiple sclerosis	Observational	–	Not applicable	April 2023 (estimated)
NCT03691077	Effect of Ocrelizumab on Brain Innate Immune Microglial Cells Activation in MS Using PET-MRI With 18F-DPA714 (INN-MS)	Recruiting	Multiple sclerosis Relapse Primary progressive multiple sclerosis	Interventional	∙ Drug: Ocrelizumab	3	September 2022 (estimated)
NCT04230174	Effect of Ocrelizumab on Neuroinflammation in Multiple Sclerosis as Measured by 11C-PBR28 MR-PET Imaging of Microglia Activation	Not yet recruiting	Multiple sclerosis	Interventional	∙ Drug: 11C-PBR28	4	July 2022 (estimated)
NCT04546698	5-HT7 Receptor Implication in Inflammatory Mechanisms in Multiple Sclerosis (5-HTSEP)	Recruiting	Multiple sclerosis Acute relapsing multiple sclerosis	Observational	∙ Other: Blood sampling	Not applicable	March 2022 (estimated)
NCT04510220	9-Month Study to Assess the Efficacy of Ofatumumab on Microglia in Patients With Relapsing Forms of Multiple Sclerosis	Recruiting	Relapsing multiple sclerosis	Interventional	∙ Drug: Ofatumumab ∙ Drug: [F-18]PBR06	3	December 31, 2021 (estimated)
NCT03759522	Assessment of Neuroinflammation in Central Inflammatory Disorders Using [F-18]DPA-714 (DPA-714)	Recruiting	Fibromyalgia Chronic fatigue syndrome Multiple sclerosis	Interventional	∙ Drug: DPA-714 PET/MRI	1	November 1, 2021 (estimated)
NCT02207075	Measuring Active Microglia in Progressive Multiple Sclerosis	Active, not recruiting	Secondary progressive multiple sclerosis	Observational	∙ Drug: [C11]PK-1195 PET scan	Not applicable	December 2020 (estimated)
NCT02446886	Adrenocorticotropic Hormone (ACTH) Effects on Myelination in Subjects With MS	Terminated	Multiple sclerosis	Interventional	∙ Drug: Adrenocorticotropic hormone (ACTH) gel (H.P. Acthar)^®^	4	June 2020
NCT03193086	The Effect of Alemtuzumab on the Blood-Brain-Barrier and the Brain’s Metabolism in Multiple Sclerosis Patients	Unknown	Multiple sclerosis	Observational	∙ Drug: Alemtuzumab	Not applicable	March 31, 2020 (estimated)
NCT02305264	Imaging of Intracerebral Inflammation in MS (INFLASEP)	Unknown	Relapsing-remitting multiple sclerosis Secondary progressive multiple sclerosis Primary progressive multiple sclerosis	Interventional	∙ Drug: 18F-DPA-714 and 18F-FDG	Not applicable	February 2018
NCT01517282	Phase Ib Study to Evaluate MOR103 in Multiple Sclerosis	Completed	Multiple sclerosis	Interventional	∙ Biological: MOR103 ∙ Other: Placebo	1 - 2	February 2014
NCT00861172	Longitudinal (Weekly) Follow-Up of Active Plaques in Multiple Sclerosis With 3 Teslas Multi-Modality MRI Using Diffusion, Perfusion, Venography and Proton Spectroscopy (IRM 3T-SEP)	Completed	Lesions Multiple sclerosis	Interventional	∙ Procedure: 3T MR scanner	Not applicable	July 2012

**TABLE 2:** Registered clinical trials on https://clinicaltrials.gov focused on *microglia* and *Alzheimer disease*, *Parkinson disease*, and *amyotrophic lateral sclerosis*.

NCT number	Title	Status	Conditions	Study type	Interventions	Phase	Study completion date
NCT04840979	Discovery and Validation of Genetic Variants Affecting Microglial Activation in Alzheimer’s Disease	Recruiting	Alzheimer’s disease	Interventional	∙ Drug: 11C-ER176	2	December 2026 (estimated)
NCT04795466	Study of the Efficacy and Safety of Various Anti-inflammatory Agents in Participants With Mild Cognitive Impairment or Mild Alzheimer’s Disease	Not yet recruiting	Mild cognitive impairment	Interventional	∙ Biological: Canakinumab	2	July 3, 2023 (estimated)
			Alzheimer’s disease		∙ Other: Placebo		
NCT03645226	Gut Microbiota Across Early Stages of Synucleinopathy: From High-Risk Relatives, REM Sleep Behavior Disorder to Early Parkinson’s Disease	Recruiting	REM sleep behavior disorder	Observational	Diagnostic test: Colonoscopy	Not applicable	March 29, 2023
NCT03702816	The Relationship Between Neuropsychological Testing and MRI, PET and COBRE - Project 1: AIM 2 (GE-180)	Enrolling by invitation	Alzheimer’s disease	Interventional		2	May 31, 2022 (estimated)
			Parkinson’s disease		∙ Drug: GE180 PET scan		
			Inflammation				
NCT03457493	TSPO-PET for Neuroinflammation in Parkinson’s Disease	Recruiting	Parkinson’s disease	Interventional	∙ Drug: DPA-714-PET/MRI	1–2	March 2022 (estimated)
NCT04066244	Study of Safety and of the Mechanism of BLZ945 in ALS Patients	Recruiting	Amyotrophic lateral sclerosis	Interventional	∙ Drug: BLZ945	2	March 4, 2022 (estimated)
NCT04057807	Peripheral Benzodiazepine Receptors (PBR28) Brain PET Imaging With Lipopolysaccharide Challenge for the Study of Microglia Function in Alzheimer’s Disease	Recruiting	Alzheimer’s disease	Interventional	∙ Drug: LPS	Early 1	January 2022 (estimated)
NCT04559828	Attenuation of Inflammatory Processes Associated With Alzheimer’s Disease After Consumption of Pomace Olive Oil (ORIVA2)	Recruiting	Alzheimer’s disease	Interventional	∙ Other: Experimental meal	Not applicable	February 28, 2021 (estimated)
NCT02831283	Imaging Inflammation in Alzheimer’s Disease	Enrolling by invitation	Alzheimer’s disease	Interventional	∙ Drug: 11C-PBR28, 18F-Florbetaben	2	June 2021 (estimated)
					∙ Procedure: Lumbar puncture (optional)		
NCT02714036	A Biomarker Study to Evaluate MN-166 (Ibudilast) in Subjects With Amyotrophic Literal Sclerosis (ALS)	Completed	Amyotrophic lateral sclerosis	Interventional	∙ Drug: Ibudilast	1 - 2	June 30, 2020 (estimated)
NCT03412604	Effect of Modulating Gamma Oscillations Using tACS	Completed	Alzheimer’s disease	Interventional	∙ Device: Transcranial alternating current stimulation (tACS)	Not applicable	September 15, 2019
NCT03918616	P2×7 Receptor, Inflammation and Neurodegenerative Diseases (NeuroInfiam)	Completed	Neurodegenerative diseases	Observational	∙ Drug: Memantine, dopamine receptor-agonists	Not applicable	March 30, 2019
NCT03548883	Examining Neuroinflammation in AlzHeimer’s (ENHANCE)	Unknown	Alzheimer’s disease, early-onset	Observational	–	Not applicable	December 2018 (estimated)

Twenty three trials were dedicated to AD, PD, ALS, or frontotemporal dementia (FTD) patients. Among these trials, nine were designed on AD patients, seven on ALS/FTD patients, and seven on PD patients. There are even four trials dedicated to patients with mild cognitive impairment or memory impairment. The analysis of trial characteristics shows that eight trials that are designed on AD, six on ALS/FTD, and four on PD are interventional clinical trials (Table 2).

We classified these trials according to the country they were conducted in. Overall, 18 out of 39 are conducted in the United States, 7 in France, 4 in Finland, 3 in Germany, 1 in Italy, 1 in Israel, 1 in Sweden, 1 in Canada, 1 in China, 1 in Denmark, and 1 in Spain. Few trials are multi-center studies and enroll patients from different countries. Therefore, the results indicate that most studies are performed in the United States and many of them are focused to study MS and AD, especially regarding interventional studies (Tables 1, 2 show the more recent trials).

Six studies are completed but only two showed results. Among studies that are completed, one is dedicated to investigating the effects of cyclophosphamide in AD patients and therefore to study inflammation and immune responses that involve microglia and astrocytes in this disorder. The goal of the trial is to reach immunomodulation in patients and to reduce the release of toxic species that can affect neuronal survival.

A study on PD patients aims to investigate the effects of 8 weeks of treatment of AZD3241 (AstraZeneca), which is a brain-penetrant myeloperoxidase inhibitor. Myeloperoxidase is a reactive oxygen-generating enzyme that is highly expressed by microglia. AZD3241 should act on myeloperoxidase, inhibiting the self-perpetuating cycle of the oxidative toxic inflammatory process and slowing down the progression of neurodegeneration. The trial follows a small phase 2a randomized placebo-controlled multicenter PET study that reported a reduction of TSPO in PD patients treated with this drug (Jucaite et al., 2015).

One trial is an observational study that aims to investigate plasma lipopolysaccharide-binding protein (LPB) levels in PD patients and controls. The final goal is to identify biomarkers for PD diagnosis. The general concept behind the study is that inflammation plays a major role in neurodegeneration associated with PD. Microglia can be trigger by different signals and upon activation it can release pro-inflammatory cytokines in both the striatum and substantia nigra, contributing to the neurodegeneration. Lipopolysaccharide can induce neurotoxicity in humans that are exposed to this endotoxin through the intestinal tract. Indeed, LPS could gain access to the brain via the enteric nervous system, ascend through the basal mid- and forebrain and finally reach the cerebral cortex. Thus, the study of LBP could reveal a new biomarker for PD across a spectrum of disease severity.

An observational trial on P2RX7 inflammatory complex on cells derived from blood samples attempts to capture the expression of these proteins in a large group of subjects with PD and with AD. The goal of the trial is to observe changes from the baseline of these proteins along with the progression of the disease. P2RX7 is activated by ATP released by dying neurons and consequently activates NLRP3, inflammasomes, and pro-inflammatory interleukin secretion (Di Virgilio et al., 2018).

A completed trial reporting results is the multi-center, open-label study of MN-166 (Ibudilast) in subjects with ALS. Ibudilast crosses the BBB after oral administration and inhibits macrophage migration inhibitory factor (MIF) and phosphodiesterases 3, 4, 10, and 11 (Gibson et al., 2006; Cho et al., 2010). Ibudilast suppresses neuronal cell death induced by microglia activation *in vitro* (Mizuno et al., 2004). Interestingly, the administration of this small molecule in myelin basic protein (MBP)-induced EAE exerts a significant amelioration of clinical and pathological outcomes. Although, such amelioration is lost in animals receiving Ibudilast according to a therapeutic intervention (Fujimoto et al., 1999). The use of Ibudilast in patients with primary or secondary progressive MS in phase 2 randomized trial, nonetheless induced a clear reduction of brain atrophy during disease progression, although patients experienced several side effects (Fox et al., 2018). However, the use of Ibudilast in ALS patients did not produce any significant reductions in motor cortical glial activation assessed by TSPO measurements as well as did not reduce CNS neuroaxonal loss, measured by serum neurofilament light (NfL) (Babu et al., 2021).

Another study that reports results is focused to evaluate the effects of multifocal transcranial alternating current stimulation (tACS) in AD patients with amyloid-positive PET and MRI information. Investigators enrolled 10 participants with AD and cerebral amyloid deposits by PET scan. They finally enrolled five persons (age 72 ± 12 years) that were eligible for the trial. The final goal of the trial is to induce microglial activation and to decrease cerebral amyloid and tau depositions. Each participant received a PET scan at the baseline and then 20 sessions of tACS intervention on consecutive weekdays. The assessment of the effect of stimulation on microglia activation, amyloid deposition, and tau deposition constitute a primary outcome measure. Investigators compared PET scans acquired before and after the tACS sections and calculated the level of proteins in the brain. They report a reduction in the levels of proteins in the brain post tACS intervention. Although, this trial involved few subjects and lacked the group of control, the results provided by investigators are suggestive of a potential treatment for AD.

The modulation of microglia through the small molecule BLZ945 is another attractive strategy that is ongoing in a trial on ALS subjects. This clinical trial is an exploratory, open-label study of multiple doses of BLZ945, which is a CSF1R kinase inhibitor (Pyonteck et al., 2013; Krauser et al., 2015) in participants with ALS. The inhibition of Csf1R has been tested in different animal models of neuroinflammatory disorders. For example, Csf1R inhibitor depletes microglia in the brain and significantly improves remyelination in the cortex and striatum of mice receiving cuprizone intoxication, although it does not alter the disease course of EAE mice (Beckmann et al., 2018). In ALS mice Csf1R levels are dramatically increased in microglia when compared with microglia from healthy mice. The inhibition of this receptor with a small molecule (GW2580) exerts a substantial impairment of microglia proliferation reducing the number of CD68+ activated microglia in the spinal cord. Mice receiving Csf1R inhibition slowed the progression of the disease supporting the notion that the activation of CSF1R signaling in ALS is relevant for the pathobiology of the disease. Part of the effects elicited by Csf1R inhibition can derive from the GW2580-mediated inhibition of macrophages infiltrating nerves of ALS mice (Martinez-Muriana et al., 2016). Peripheral myeloid cells display constitutive expression of Csf1R (Sherr, 1990); they can invade nerves of ALS exerting detrimental effects (Liu et al., 2012). Thus, we can speculate that blunting the inflammation in both the central and peripheral nervous systems could result in a significant amelioration of the disease course.

## Conclusion

The complexity of the pathological mechanisms sustaining MS, AD, ALS, and PD is probably the main obstacle to develop effective therapeutic interventions. However, a long list of studies highlights the importance of neuroinflammation in virtually all neurodegenerative diseases. Among neuroinflammatory players, studies analyzing microglia activation enforce the idea that these cells could have an impact that is far to be trivial in these neurodegenerative disorders.

However, unresolved questions emerge from these studies that deal with diseased microglia, the specificity of such activation in different disorders, and finally the contribution of these cells to the pathological progression. On one hand, we know from postmortem-derived microglia that they are surprisingly heterogeneous. On the other hand, microglia activation and the release of pro-inflammatory signals in the extracellular matrix seem to be a common output in AD, PD, and ALS.

Microglia diversity in humans has been captured by single cells transcriptomics and goes beyond the classic concept of M1/2 phenotypes. However, what is missing is the trajectory that these cells acquire during the initial phase as well as during the progressive phase of these disorders. This aspect is relevant if we want to develop pharmacological tools able to modulate microglia in patients. A large body of experimental evidence obtained in animal models suggests that microglia might be an immunological entry point for therapeutics interventions aimed to slow down the progression of neurodegeneration. However, general limitations of animal models impose to proceed with caution in the interpretation of these results. Indeed, some alterations that are featuring animal models of ALS, PD, and AD do not overlap with pathological alterations observed in patients. Moreover, some models failed to recapitulate the entire spectrum of individual pathology, thus providing an incomplete picture of the degenerative processes.

With these limitations, the generation of more robust and reliable models is an imperative requirement for scientists dealing with neurodegenerative diseases. Possibly, the use of iPSCs-derived cultures as well as the generation of organoids incorporating microglia could help to investigate, at least some of, these fundamental questions (Fagerlund et al., bioRxiv preprint^3^). Indeed, analyzing cell subset diversity within the diseased brain using new experimental approaches (Alon et al., 2021) as well as the RNA characteristics might reveal unexplored pathogenic mechanisms.

Addressing these limitations is still a fundamental issue to be further explored to obtain a final picture of the precise role of microglia in neurodegenerative disorders. Using large datasets will be certainly instrumental to properly set up and develop new therapeutic strategies for the treatment of these devastating and irreversible neurological diseases.

## Author Contributions

LM wrote the manuscript with support from AV and GM. All authors contributed to the article and approved the submitted version.

## Conflict of Interest

The authors declare that the research was conducted in the absence of any commercial or financial relationships that could be construed as a potential conflict of interest.

## Publisher’s Note

All claims expressed in this article are solely those of the authors and do not necessarily represent those of their affiliated organizations, or those of the publisher, the editors and the reviewers. Any product that may be evaluated in this article, or claim that may be made by its manufacturer, is not guaranteed or endorsed by the publisher.

## Footnotes

1
https://doi.org/10.1101/2020.09.28.20202812

2
https://clinicaltrials.gov/

3
https://doi.org/10.1101/2020.12.08.416388
